# Right Ventricular Global Longitudinal Strain and 30‐Day Mortality in Intermediate‐Risk Pulmonary Embolism: An External Validation Study

**DOI:** 10.1111/echo.70612

**Published:** 2026-09-10

**Authors:** Shunsuke Eguchi, Ayumi Eguchi, Yoshiyuki Orihara, Michael Pfeiffer, Brandon Peterson, John Boehmer, Robert Biederman, Ryan Wilson

**Affiliations:** ^1^ Heart and Vascular Institute Pennsylvania State University College of Medicine Hershey Pennsylvania USA; ^2^ West Virginia University Morgantown West Virginia USA; ^3^ Roper/Saint Francis Hospital Charleston South Carolina USA; ^4^ Medical University of South Carolina Charleston South Carolina USA; ^5^ Division of Cardiology Allegheny General Hospital Pittsburgh Pennsylvania USA

**Keywords:** prognosis, right ventricular dysfunction, right ventricular strain, risk stratification, speckle‐tracking echocardiography

## Abstract

**Background:**

Intermediate‐risk pulmonary embolism (PE) carries substantial short‐term mortality. Our group previously demonstrated that right ventricular global longitudinal strain (RVGLS) predicted 30‐day mortality in a single‐center cohort using data collected before contemporary guideline updates, identifying a cutoff value of 17.7%. However, its prognostic value in contemporary external cohorts remains uncertain. We aimed to externally validate the association between RVGLS and 30‐day mortality in an independent cohort.

**Methods:**

We performed a retrospective cohort study of 119 patients with intermediate‐risk PE treated between 2019 and 2022 at an independent institution. The primary outcome was all‐cause 30‐day mortality. Clinical and echocardiographic parameters were compared between survivors and non‐survivors. Receiver operating characteristic and Kaplan–Meier analyses were used to assess prognostic performance.

**Results:**

The mean age was 66.1 years, and 48.7% were female. Six patients (5.0%) died within 30 days. RV strain analysis was feasible in 110 patients (92.4%). Non‐survivors had lower RVGLS (17.3% vs. 19.6%, *p* = 0.013). In univariable analysis, RVGLS was associated with mortality (odds ratio 0.567, *p* = 0.033). RVGLS demonstrated discriminatory performance for 30‐day mortality, with an area under the curve of 0.802. Using the previously established cutoff of 17.7%, patients with reduced RVGLS had higher 30‐day mortality (Log‐rank *p* = 0.042).

**Conclusions:**

In this external cohort, RVGLS ≤ 17.7% was associated with increased 30‐day mortality, supporting the prognostic value of the previously established threshold beyond the original derivation cohort.

## Graphical abstract

Reduced right ventricular global longitudinal strain (RVGLS) was associated with increased 30‐day mortality in an external cohort of patients with intermediate‐risk pulmonary embolism. RVGLS demonstrated discriminative ability (AUC 0.802), and the previously established 17.7% cutoff was associated with differences in 30‐day survival, supporting its potential prognostic value for risk stratification.

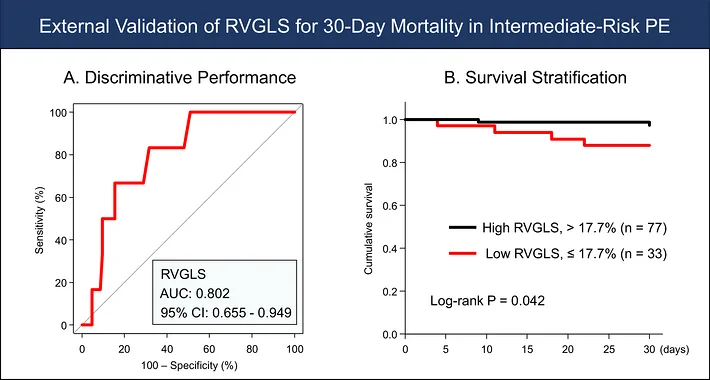

**Abbreviations**
AUC: area under the curve
PE: pulmonary embolism
ROC: receiver operating characteristic
RVFWS: right ventricular free wall strain
RVGLS: right ventricular global longitudinal strain

## 1 Introduction

Acute pulmonary embolism (PE) remains a major contributor to the global disease burden and is associated with substantial short‐term mortality [1, 2]. Patients with acute PE are stratified into low‐, intermediate‐, and high‐risk categories based on hemodynamic status, cardiac biomarker levels, and evidence of right ventricular (RV) dysfunction on cardiac imaging [3, 4]. Patients with intermediate‐risk PE are defined as being hemodynamically stable at presentation but with evidence of RV dysfunction or elevated cardiac biomarkers [3]. This population carries a significant short‐term mortality risk ranging from 3% to 15% [4, 5, 6].

Echocardiography plays an important role in evaluating RV size and function in patients presenting with PE and in identifying the presence of RV dysfunction [3]. Conventional echocardiographic parameters such as right‐to‐left ventricular end‐diastolic diameter ratio (RV/LV ratio), tricuspid annular plane systolic excursion (TAPSE), and tissue Doppler imaging‐derived right ventricular systolic velocity (RV S’) have been incorporated into risk assessment strategies [3, 7, 8, 9]. However, these conventional parameters either rely on linear measurements of ventricular size or primarily reflect basal longitudinal motion of the RV free wall and may not fully represent global RV systolic function [10, 11, 12].

Two‐dimensional speckle‐tracking strain imaging overcomes some of the limitations and pitfalls of conventional parameters and enables quantitative assessment of myocardial deformation. It has been shown to detect subclinical RV dysfunction and provide additional prognostic information beyond conventional echocardiographic parameters [11, 13]. In our previous single‐center study of patients with intermediate‐risk PE between 2010 and 2018, RV global longitudinal strain (RVGLS) was strongly associated with 30‐day mortality, and a cutoff value of 17.7% provided high sensitivity for 30‐day mortality [14]. The addition of RVGLS to conventional parameters provided incremental prognostic value [15].

The 2019 European Society of Cardiology guidelines for the diagnosis and management of acute PE emphasized risk stratification and contemporary management strategies [3]. These changes in clinical practice may influence the prognostic performance of imaging markers evaluated in earlier cohorts managed under different care settings. However, the prognostic value of RVGLS in external cohorts remains uncertain. Therefore, we conducted an external validation study to determine whether RVGLS and the previously established cutoff value of 17.7% from a single‐center derivation cohort retain prognostic relevance in patients with intermediate‐risk PE.

## 2 Methods

### 2.1 Study Design and Population

This was a retrospective cohort study of consecutive adult patients with intermediate‐risk PE admitted to Allegheny General Hospital between 2019 and 2022. This study was approved by the Institutional Review Boards of Allegheny General Hospital and Pennsylvania State University, and the requirement for informed consent was waived due to the retrospective nature of the study.

Intermediate‐risk PE was defined as acute PE without systemic hypotension (systolic blood pressure [BP] < 90 mmHg) and with objective evidence of RV dysfunction or myocardial injury, consistent with contemporary guideline‐based definitions [3]. Patients who required emergent thrombolysis, surgical embolectomy, or vasopressor support prior to echocardiographic evaluation were excluded. The primary outcome was all‐cause mortality within 30 days of PE diagnosis. Thirty‐day mortality and the date of death were determined by review of the electronic medical record. All survivors had follow‐up documentation confirming that they were alive at least 30 days after PE diagnosis. Clinical data, including demographics, comorbidities, and vital signs at presentation, were collected from the electronic medical record.

For comparison, data from our previously reported derivation cohort of patients with intermediate‐risk PE at Pennsylvania State University between 2010 and 2018 (*n* = 251) were used [14, 15].

### 2.2 Echocardiographic Acquisition and Analysis

All transthoracic echocardiograms were obtained as part of routine clinical practice and were performed within 48 h of initial PE diagnosis. All studies were analyzed at the echocardiographic core laboratory (Pennsylvania State University College of Medicine, Hershey, PA, USA) by experienced readers blinded to clinical outcomes and baseline clinical data.

Conventional echocardiographic parameters were measured according to current guideline recommendations [16, 17]. RV and LV end‐diastolic diameters used to calculate the RV/LV ratio were measured from the standard apical four‐chamber view at end‐diastole, defined as the frame in which ventricular chamber size was the largest, at the level of the tricuspid and mitral valve leaflet tips, consistent with previously published echocardiographic methods [15, 18]. RV fractional area change (RVFAC), TAPSE, and RV S’ were measured using RV‐focused apical four‐chamber views to optimize visualization of the RV free wall. RV‐pulmonary artery (RV‐PA) coupling indices were calculated as ratios of RV systolic function parameters to systolic pulmonary artery pressure (SPAP), including RVFAC/SPAP, TAPSE/SPAP, and RV S’/SPAP [17]. SPAP was estimated using the simplified Bernoulli equation from the peak tricuspid regurgitation velocity, with right atrial (RA) pressure estimated based on inferior vena cava diameter and respiratory variation, in accordance with established echocardiographic guidelines [16, 17].

RV strain analysis was performed using vendor‐independent speckle‐tracking software (AutoStrain RV, version TTA 2.51.02, TOMTEC Imaging Systems GmbH, Munich, Germany) within the Philips Ultrasound Workspace platform (Philips Healthcare, Andover, MA, USA). RVGLS was calculated from RV‐focused apical four‐chamber views as the average peak systolic longitudinal strain of six RV segments (three free‐wall and three septal segments). RV free wall strain (RVFWS) was derived as the average of the three RV free‐wall segments. RA strain analysis was performed using the 2D Cardiac Performance Analysis application (TOMTEC Imaging Systems). RA strain was defined as peak longitudinal reservoir strain during ventricular systole. Studies with inadequate tracking quality were excluded from strain analysis. Strain values were recorded and reported as absolute values to facilitate clinical interpretation and consistency with prior reports [14, 15].

### 2.3 Statistical Analysis

Continuous variables were presented as mean ± standard deviation and compared using unpaired t‐tests or Mann‐Whitney U tests. Categorical variables were presented as counts and percentages and compared using Fisher's exact test. Comparisons were performed between 30‐day survivors and non‐survivors, as well as between the derivation and external cohorts using these methods. Univariable logistic regression analysis was performed to identify variables associated with 30‐day mortality. Odds ratios (OR) were presented per unit increase for each continuous variable. For RV/LV ratio and RV‐PA coupling indices (RVFAC/SPAP, TAPSE/SPAP, and RV S’/SPAP), ORs were presented per 0.1 increase to improve clinical interpretability. Receiver operating characteristic (ROC) curve analysis was used to evaluate the discriminative ability of RVGLS for 30‐day mortality. The previously reported cutoff value of 17.7% was applied to assess its performance in this cohort. The optimal cutoff value was determined using the Youden index as an exploratory analysis [19]. Kaplan–Meier survival curves were constructed using the predefined cutoff value of 17.7% and compared using the log‐rank test. Analyses were performed using available data without imputation for missing values. A two‐sided *p*‐value < 0.05 was considered statistically significant. Statistical analyses were performed with SPSS software (IBM Corp., Armonk, NY, USA, version 30.0.0.0).

## 3 Results

### 3.1 Baseline Clinical Characteristics

A total of 119 patients with intermediate‐risk PE were included, of whom 6 patients (5.0%) died within 30 days. Baseline clinical characteristics are summarized in Table 1. The mean age was 66.1 ± 14.0 years, and 58 patients (49%) were female. The prevalence of baseline comorbidities was similar between survivors and non‐survivors, with no statistically significant differences observed. Among vital signs at presentation, diastolic blood pressure was significantly lower in non‐survivors compared with survivors (59.7 ± 29.5 mmHg vs. 80.8 ± 14.8 mmHg, *p* = 0.025). No other baseline clinical characteristics differed significantly between the two groups.

**TABLE 1:** Baseline clinical characteristics according to 30‐day survival status in patients with intermediate‐risk PE.

	All	30‐day survivor	30‐day non‐survivor	*p*‐value
Subjects, *n*	119	113	6	
Age, years	66.1 ± 14.0	66.0 ± 14.2	68.5 ± 9.8	0.693
Female, *n* (%)	58 (49)	55 (49)	3 (50)	1.000
Race, Black, *n* (%)	20 (17)	20 (18)	0 (0)	0.588
Hypertension, *n* (%)	98 (82)	93 (82)	5 (83)	1.000
DM, *n* (%)	45 (38)	42 (37)	3 (50)	0.672
HL, *n* (%)	81 (68)	79 (70)	2 (33)	0.081
CHF, *n* (%)	34 (29)	34 (30)	0 (0)	0.181
CAD, *n* (%)	33 (28)	33 (29)	0 (0)	0.185
AF, *n* (%)	25 (21)	25 (22)	0 (0)	0.341
CVA, *n* (%)	27 (23)	25 (22)	2 (33)	0.617
OSA, *n* (%)	47 (39)	47 (42)	0 (0)	0.080
CKD, *n* (%)	26 (22)	24 (21)	2 (33)	0.611
ESRD, *n* (%)	2 (2)	2 (2)	0 (0)	1.000
Vital sign on presentation				
Heart rate, beats/min	90.6 ± 14.9	90.7 ± 15.1	87.7 ± 10.2	0.524
Systolic BP, mmHg	131.8 ± 22.9	132.3 ± 22.1	121.5 ± 35.5	0.280
Diastolic BP, mmHg	79.8 ± 16.3	80.8 ± 14.8	59.7 ± 29.5	0.025

*Note*: Data are presented as mean ± SD or number (%), as appropriate.

Abbreviations: AF, atrial fibrillation; BP, blood pressure; CAD, coronary artery disease; CHF, chronic heart failure; CKD, chronic kidney disease; CVA, cerebrovascular accident; DM, diabetes mellitus; ESRD, end‐stage renal disease; HL, hyperlipidemia; OSA, obstructive sleep apnea.

Comparison of baseline clinical characteristics between the derivation and external cohorts is summarized in Table S1. Compared with the derivation cohort, the external cohort had a higher proportion of Black patients and a higher prevalence of hypertension and chronic heart failure. In addition, heart rate was significantly lower in the external cohort, while systolic and diastolic blood pressures were similar between cohorts.

### 3.2 Echocardiographic Parameters and Strain Analysis

Echocardiographic parameters stratified by 30‐day survival status are summarized in Table 2. Image quality was sufficient to perform RVGLS analysis in 110 patients (92.4%) and RA strain analysis in 105 patients (88.2%). Among conventional echocardiographic parameters, there were no statistically significant differences between survivors and non‐survivors. However, RV/LV ratio (1.05 ± 0.08 vs. 0.95 ± 0.14, *p* = 0.058) and TAPSE (14.2 ± 3.06 mm vs. 17.3 ± 4.19 mm, *p* = 0.087) showed trends toward higher RV/LV ratio and lower TAPSE in non‐survivors. RV‐PA coupling indices (RVFAC/SPAP, TAPSE/SPAP, and RV S’/SPAP) did not differ significantly between survivors and non‐survivors.

**TABLE 2:** Comparison of conventional and strain echocardiographic parameters between 30‐day survivors and non‐survivors.

	All	30‐day survivor	30‐day non‐survivor	*p*‐value
Conventional parameters				
LVEF, %	61.0 ± 10.7	60.8 ± 10.9	65.9 ± 5.51	0.182
LVDd, mm	44.0 ± 5.93	44.2 ± 6.01	40.3 ± 2.34	0.062
RVDd, mm	41.6 ± 6.77	41.6 ± 6.87	42.2 ± 4.96	0.628
RV/LV ratio	0.95 ± 0.14	0.95 ± 0.14	1.05 ± 0.08	0.058
RVFAC, %	33.7 ± 6.60	33.8 ± 6.71	30.7 ± 2.89	0.216
TAPSE, mm	17.1 ± 4.19	17.3 ± 4.19	14.2 ± 3.06	0.087
RV S’, cm/sec	12.8 ± 3.35	12.8 ± 3.40	12.7 ± 2.58	0.918
SPAP, mmHg	43.1 ± 16.3	43.6 ± 16.2	35.0 ± 17.6	0.286
RVFAC/SPAP, %/mmHg	0.98 ± 0.65	0.97 ± 0.64	1.15 ± 0.71	0.499
TAPSE/SPAP, mm/mmHg	0.50 ± 0.32	0.50 ± 0.32	0.50 ± 0.25	0.787
RV S’/SPAP, cm/s·mmHg	0.37 ± 0.24	0.36 ± 0.24	0.48 ± 0.32	0.267
Strain parameters				
RVGLS, % ^a^	19.5 ± 2.61	19.6 ± 2.61	17.3 ± 1.20	0.013
RVFWS, % ^a^	21.4 ± 3.77	21.5 ± 3.80	19.7 ± 2.82	0.239
RA strain, % ^b^	31.0 ± 6.73	31.4 ± 6.69	25.8 ± 5.60	0.040

*Note*: Data are presented as mean ± SD. RV strain parameters are presented as absolute values.

Abbreviations: LVDd, left ventricular end‐diastolic diameter; LVEF, left ventricular ejection fraction; RA strain, right atrial strain; RVDd, right ventricular end‐diastolic diameter; RVFAC, right ventricular fractional area change; RVFWS, right ventricular free wall strain; RVGLS, right ventricular global longitudinal strain; RV/LV ratio, right‐to‐left ventricular end‐diastolic diameter ratio; RV S’, tissue Doppler imaging‐derived right ventricular systolic velocity; SPAP, systolic pulmonary artery pressure; TAPSE, tricuspid annular plane systolic excursion.

a Available for 110 patients (92.4 %) in the entire cohort.

b Available for 105 patients (88.2 %) in the entire cohort.

**FIGURE 1:**
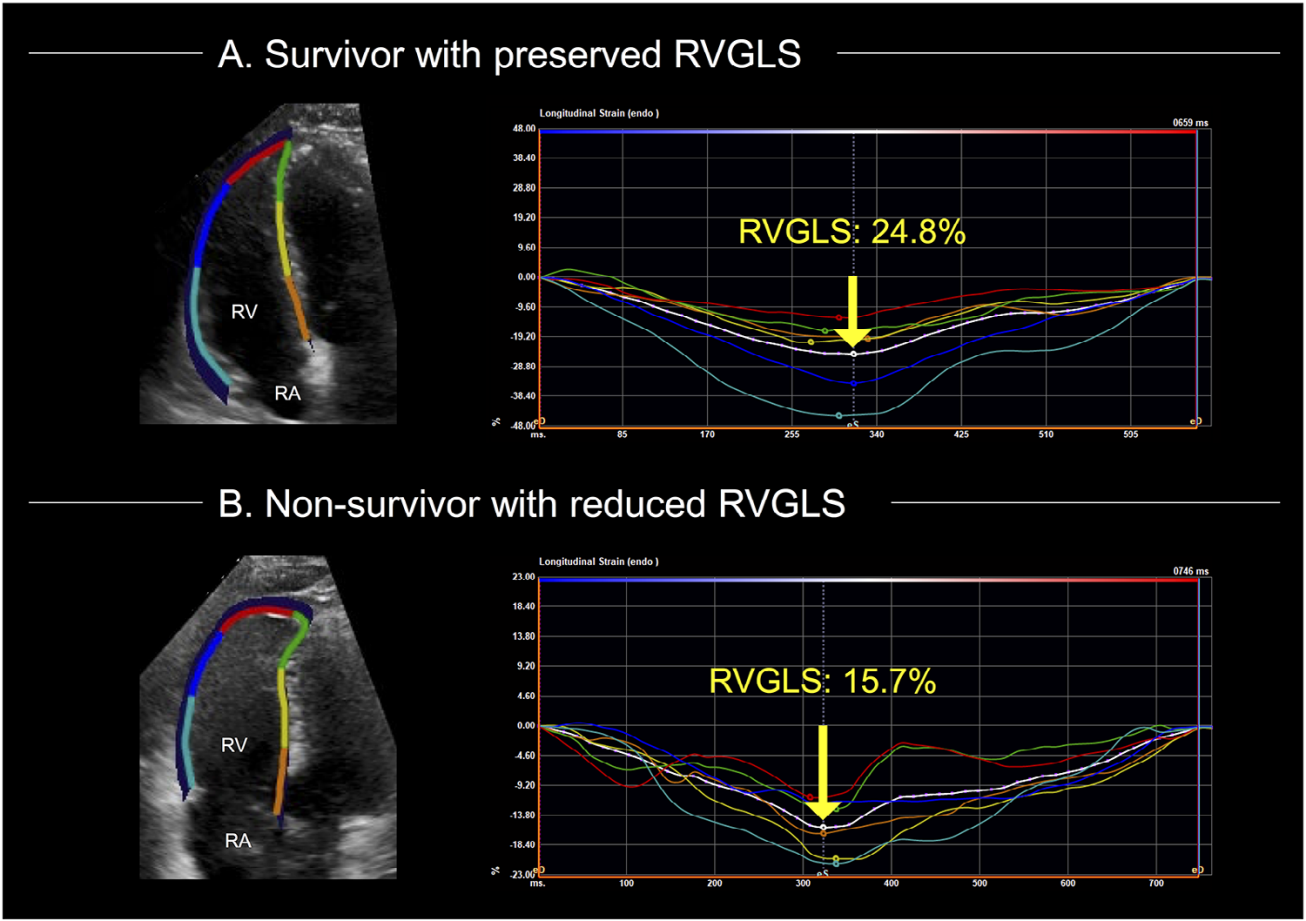
Representative baseline RVGLS in a 30‐day survivor and non‐survivor. RV‐focused apical four‐chamber views with two‐dimensional speckle‐tracking analysis are shown. A representative 30‐day survivor demonstrates preserved RVGLS (24.8%), whereas a representative 30‐day non‐survivor shows reduced RVGLS (15.7%). Strain values are presented as absolute values. RV, right ventricle; RVGLS, right ventricular global longitudinal strain.

Among strain parameters, RVGLS was significantly lower in non‐survivors compared with survivors (17.3 ± 1.20% vs. 19.6 ± 2.61%, *p* = 0.013). RA strain was also significantly reduced in non‐survivors (25.8 ± 5.60% vs. 31.4 ± 6.69%, *p* = 0.040). In contrast, RV free wall strain did not differ significantly between groups (*p* = 0.239). Representative examples of RV strain analysis in a 30‐day survivor and non‐survivor are shown in Figure 1.

Comparison of echocardiographic parameters between the derivation and external cohorts is also shown in Table S1. LVDd, RVDd, and TAPSE were significantly lower in the external cohort, whereas other conventional and strain parameters, including RVGLS, did not differ significantly between cohorts.

### 3.3 Prognostic Performance of RVGLS

In univariable logistic regression analysis (Table 3), RVGLS was significantly associated with 30‐day mortality (OR 0.567, 95% confidence interval [CI] 0.336–0.955, *p* = 0.033). RA strain was also associated with mortality (OR 0.865, 95% CI 0.751–0.997, *p* = 0.046). Among clinical variables, lower diastolic BP was associated with mortality (OR 0.936, 95% CI 0.891–0.983, *p* = 0.008). Conventional echocardiographic parameters, including LVEF, RV/LV ratio, RVFAC, and TAPSE, were not significantly associated with 30‐day mortality in this patient cohort. Similarly, none of the RV‐PA coupling indices (RVFAC/SPAP, TAPSE/SPAP, and RV S’/SPAP) were significantly associated with 30‐day mortality.

**TABLE 3:** Univariable logistic regression analysis of clinical and echocardiographic variables for 30‐day mortality

Indicators	OR	95 % CI	*p*‐value
Vital sign on presentation			
Heart rate, beats/min	0.986	0.933–1.040	0.624
Systolic BP, mmHg	0.979	0.942–1.020	0.264
Diastolic BP, mmHg	0.936	0.891–0.983	0.008
Echocardiographic conventional parameters			
LVEF, %	1.060	0.957–1.180	0.254
LVDd, mm	0.880	0.751–1.030	0.116
RVDd, mm	1.010	0.899–1.140	0.843
RV/LV ratio ^a^	1.380	0.787–2.420	0.261
RVFAC, %	0.920	0.797–1.060	0.253
TAPSE, mm	0.786	0.603–1.030	0.076
RV S’, cm/sec	0.985	0.766–1.270	0.907
SPAP, mmHg	0.965	0.913–1.020	0.216
RVFAC/SPAP, %/mmHg ^a^	1.040	0.933–1.150	0.511
TAPSE/SPAP, mm/mmHg ^a^	0.997	0.767–1.300	0.982
RV S’/SPAP, cm/s·mmHg ^a^	1.170	0.892–1.540	0.255
Strain parameters			
RVGLS, %	0.567	0.336–0.955	0.033
RVFWS, %	0.845	0.631–1.130	0.257
RA strain, %	0.865	0.751–0.997	0.046

*Note*: RV strain parameters are presented as absolute values.

Abbreviations: BP, blood pressure; CI, confidence interval, LVDd, left ventricular end‐diastolic diameter; LVEF, left ventricular ejection fraction; OR, odds ratio; RA strain, right atrial strain; RVDd, right ventricular end‐diastolic diameter; RVFAC, right ventricular fractional area change; RVFWS, right ventricular free wall strain; RVGLS, right ventricular global longitudinal strain; RV/LV ratio, right‐to‐left ventricular end‐diastolic diameter ratio; RV S’, tissue Doppler imaging‐derived right ventricular systolic velocity; SPAP, systolic pulmonary artery pressure; TAPSE, tricuspid annular plane systolic excursion.

a ORs are presented per 0.1 increase.

**FIGURE 2:**
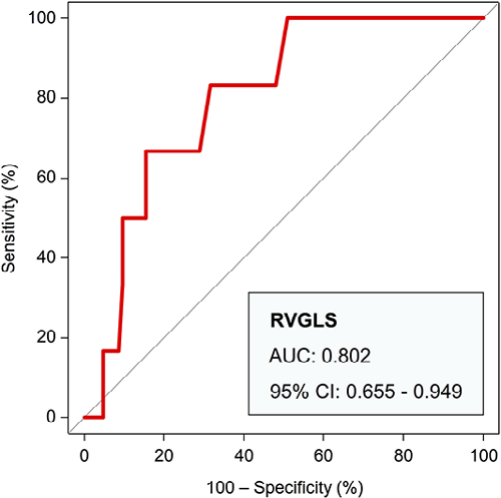
ROC curve of RVGLS for 30‐day mortality. The ROC curve illustrates the discriminative performance of RVGLS for 30‐day mortality. The AUC was 0.802 (95% CI: 0.655–0.949). Using the previously established cutoff value of 17.7%, the sensitivity and specificity for identifying patients at increased risk of 30‐day mortality were 66.7% and 72.1%, respectively. AUC, area under the curve; CI, confidence interval; ROC, receiver operating characteristic; RVGLS, right ventricular global longitudinal strain.

ROC curve analysis demonstrated that RVGLS had discriminative ability for 30‐day mortality, with an area under the curve (AUC) of 0.802 (95% CI 0.655–0.949) (Figure 2). Using the previously established cutoff value of 17.7% [14, 15], RVGLS demonstrated a sensitivity of 66.7% and a specificity of 72.1% for identifying patients who had a 30‐day mortality. The positive predictive value (PPV) was 12.1% and the negative predictive value (NPV) was 97.4%. In an exploratory analysis, the optimal RVGLS cutoff value in this external cohort was 18.0%, yielding a sensitivity of 83.3% and a specificity of 68.3%. The corresponding PPV and NPV were 13.2% and 98.6%, respectively. Diastolic BP and RA strain also demonstrated moderate discriminative ability, with AUCs of 0.773 (95% CI 0.482–1.000) and 0.751 (95% CI 0.623–0.879), respectively (Figure S1).

**FIGURE 3:**
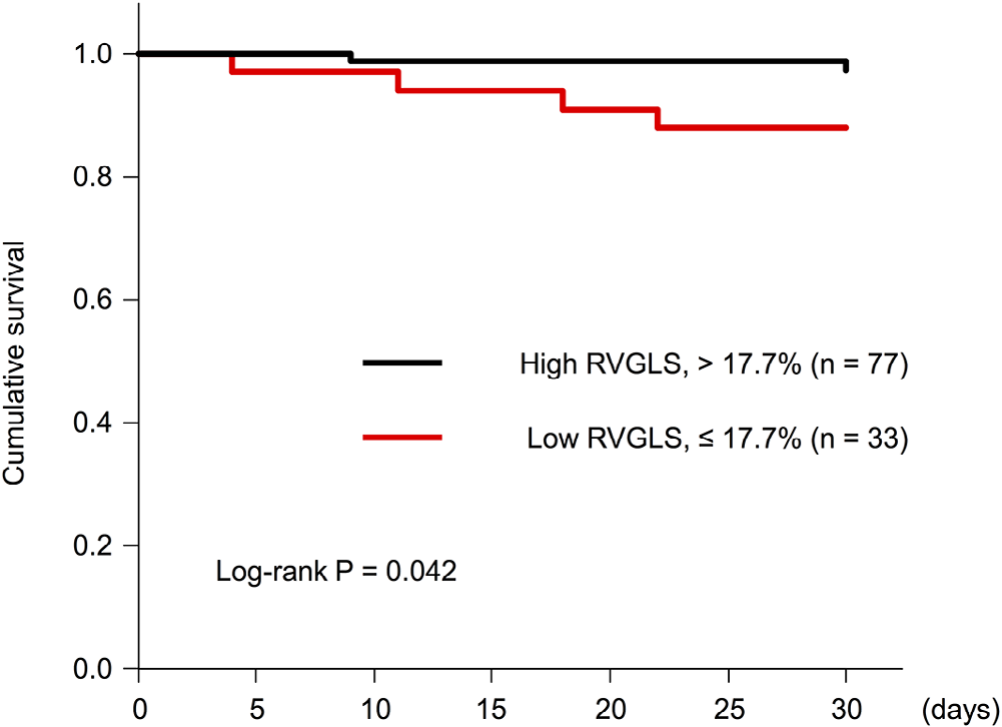
Kaplan–Meier survival curves stratified by RVGLS cutoff of 17.7%. Patients were stratified into two groups based on RVGLS ≤ 17.7% and > 17.7%. Patients with reduced RVGLS (≤ 17.7%) had significantly lower survival within 30 days compared with those with preserved RVGLS (> 17.7%) (Log‐rank *p* = 0.042). RVGLS, right ventricular global longitudinal strain.

Kaplan–Meier survival analysis further demonstrated significant separation between groups stratified by RVGLS cutoff (Figure 3). Patients with RVGLS ≤ 17.7% had significantly lower cumulative survival compared with those with RVGLS > 17.7% (Log‐rank *p* = 0.042).

Overall, the prognostic performance of RVGLS in this external cohort was consistent with that observed in the derivation cohort [14, 15]. While the AUC was slightly lower in the external cohort (0.802 vs. 0.855), RVGLS remained significantly associated with 30‐day mortality. Importantly, the previously established cutoff value of 17.7% also effectively stratified patients in the external cohort. Kaplan–Meier analysis showed a significant difference in survival between patients with RVGLS ≤ 17.7% and those with RVGLS > 17.7%, supporting the reproducibility of these results.

## 4 Discussion

This external cohort of patients with intermediate‐risk PE validates our previously reported finding that patients with decreased RVGLS at baseline have an increased 30‐day mortality. The previously established cutoff value of 17.7% maintained prognostic significance to discriminate patients at higher and lower risk for short‐term mortality. The findings of this study validate previous observations and further support RVGLS as a reproducible prognostic marker beyond the original derivation cohort.

### 4.1 Physiological Basis for the Prognostic Value of RVGLS

In the present study, RVGLS remained a significant predictor of 30‐day mortality in patients with intermediate‐risk PE, validating our earlier observations and further supporting its prognostic relevance [14, 15]. RVGLS reflects intrinsic myocardial deformation and provides an integrated assessment of global RV systolic function [20, 21]. Unlike conventional parameters such as TAPSE or RV S’, which primarily reflect basal longitudinal motion of the RV free wall and are load‐ and angle‐dependent, RVGLS integrates myocardial deformation across the entire RV myocardium, including both the free wall and interventricular septum [10, 11, 12, 22]. Strain analysis directly quantifies myocardial deformation rather than geometric change, therefore reduced strain may occur before detectable alterations in chamber size or conventional functional parameters. This characteristic allows RVGLS to detect early RV dysfunction even when conventional parameters remain within normal or borderline ranges [10, 11].

The interventricular septum also plays a critical role in RV performance because it provides mechanical coupling between the RV and LV [23]. Previous studies have demonstrated that LV contraction contributes substantially to RV systolic pressure and stroke volume through the interventricular septum [24, 25, 26]. RVGLS incorporates both RV free wall and septal deformation, allowing assessment of intrinsic RV contractility as well as RV‐LV interaction. In contrast, RVFWS evaluates deformation only within the RV free wall and may not fully capture RV‐LV interaction. This mechanistic difference may account for the observed association between RVGLS and mortality in the present study, whereas RVFWS was not associated with mortality.

Additional metrics such as RV‐PA coupling, which reflects the ability of the RV to adapt to pulmonary vascular afterload and can be approximated noninvasively using SPAP‐based indices such as TAPSE/SPAP, have been evaluated in acute PE [17, 27]. Recent observational studies have reported associations between reduced TAPSE/SPAP and early clinical deterioration or short‐term mortality [28, 29, 30]. In this external cohort, however, RVFAC/SPAP, TAPSE/SPAP, and RV S’/SPAP were not associated with 30‐day mortality. Given the limited number of mortality events, the absence of a significant association should be interpreted cautiously and does not establish that RV‐PA coupling indices lack prognostic value in patients with intermediate‐risk PE more broadly. RVGLS reflects myocardial deformation, whereas RV‐PA coupling indices relate RV systolic performance to pulmonary afterload. Whether these measures provide complementary prognostic information requires further investigation.

### 4.2 Clinical Implications of External Validation

External validation is an important step before incorporating imaging markers into routine clinical decision‐making. Our prior work demonstrated the prognostic value of RVGLS in a single‐center cohort [14, 15]. The present study externally validates these findings at an independent institution. The external validation cohort represented approximately 50% of the size of the original derivation cohort and therefore provided a relatively large independent sample for validation.

In our previously reported single‐center study, reduced RVGLS at the time of diagnosis in patients with intermediate‐risk PE was associated with increased short‐term mortality. We also identified a cutoff value of 17.7% for RVGLS that provided clinically useful risk discrimination [14, 15]. The present external validation extends these findings to a more contemporary cohort from an independent institution. The external validation cohort demonstrated a lower 30‐day mortality rate compared with the derivation cohort (5.0% vs. 12.4%), although both rates were within the range previously reported for intermediate‐risk PE [4, 5, 6]. The prognostic utility of the RVGLS threshold value established in the original study remained clinically meaningful in this external validation cohort. An exploratory analysis identified an optimal RVGLS threshold value of 18.0% in this novel patient population, which was only slightly higher than the derivation cohort threshold of 17.7%. AUC and Kaplan–Meier analysis demonstrated comparable prognostic performance across the derivation and external validation cohorts. These findings support the utility of RVGLS as a prognostic marker, with a threshold value of 17.7% identifying intermediate‐risk PE patients at higher risk for short‐term mortality. In addition, RVGLS was successfully analyzed in a vast majority of patients (91.6% in the derivation cohort; 92.4% in the validation cohort), which further supports its clinical applicability in patients presenting with acute PE. Although RVGLS and RA strain values were significantly lower in non‐survivors, the reported standard deviations indicate substantial overlap between survivors and non‐survivors. This overlap limits the clinical interpretation of an individual strain value in a single patient. The observed difference in RA strain between survivors and non‐survivors (*p* = 0.040) should be interpreted cautiously and considered exploratory. In addition, the predefined RVGLS cutoff value of 17.7% yielded a sensitivity of 66.7% and a specificity of 72.1%, with a low PPV (12.1%) and a high NPV (97.4%) that should both be interpreted in the context of the low 5.0% event rate. Accordingly, RVGLS may be considered a potential risk marker and should be interpreted together with established clinical and echocardiographic parameters.

These findings suggest that strain‐based assessment reflects intrinsic RV pathophysiology and that the prognostic value of RVGLS persists across evolving management approaches. Importantly, RVGLS may provide clinically actionable information at the time of initial echocardiographic evaluation. Identification of patients with impaired RVGLS may facilitate early risk stratification, closer clinical monitoring, and consideration of more intensive management strategies in patients at higher risk of deterioration. Conversely, preserved RVGLS may help identify lower‐risk patients who may be suitable for standard management and routine follow‐up.

### 4.3 Study Limitations

This study has several limitations. First, it was retrospective in design and conducted at a single external institution, which may limit generalizability. All echocardiographic measurements were performed at the same core laboratory that analyzed the derivation cohort, and the validation was external with respect to the patient population but not fully independent with respect to echocardiographic analysis. Second, the number of mortality events was relatively small, which restricts statistical power and precludes robust multivariable modeling. Therefore, the independent prognostic value of RVGLS beyond other clinical and echocardiographic variables could not be determined. The small number of events contributed to the wide confidence interval for the AUC, and the low event rate influenced the PPV and NPV. Third, given that this was an external validation study, we evaluated echocardiographic images from the validation cohort and had specific patient information; however, we could not personally review charts, and several clinically relevant variables, including cardiac biomarkers, clinical risk scores, cancer status, and treatment strategies, were not systematically available in the source dataset. Therefore, residual confounding cannot be excluded, and we could not determine whether RVGLS provides incremental prognostic information beyond established risk stratification. Fourth, although all echocardiograms were performed within 48 h of PE diagnosis, the interval between anticoagulation initiation and imaging was not standardized, and treatment‐related changes in RV loading conditions may have influenced the echocardiographic measurements. Fifth, although RVGLS was analyzable in 110 of 119 patients (92.4%), the inability to analyze RVGLS in 9 patients because of inadequate image quality may have introduced selection bias, particularly given the limited number of mortality events. Finally, this study was conducted before the publication of the recent 2026 multisociety guideline for acute PE, which introduced a revised risk classification framework [31]. Therefore, risk stratification in this cohort was based on the contemporaneous European Society of Cardiology guideline [3], and direct alignment with the newer classification system was not feasible.

## 5 Conclusions

In a contemporary external cohort of patients with intermediate‐risk PE, the finding of increased 30‐day mortality in patients with reduced RVGLS is validated. The previously established cutoff value of 17.7% was associated with differences in 30‐day survival. Exploratory analysis of the optimal cutoff in the validation cohort (18.0%) was similar to that of the derivation cohort. These findings support the reproducibility and potential prognostic value of RVGLS for risk stratification in intermediate‐risk PE. Further multicenter, prospective studies are warranted to refine optimal thresholds and to support broader clinical implementation in guideline‐based care.

## Funding

This study was supported by the Penn State Clinical and Translational Science Institute (CTSI)—Highmark Health Clinical and Translational Research Grant (2022‐2024).

## Supporting information




**Table S1**: Comparison of baseline clinical and echocardiographic characteristics between the derivation and external cohorts.
**Figure S1: ROC curves of diastolic BP and RA strain for 30‐day mortality**. ROC analysis demonstrating the ability of (A) diastolic BP and (B) RA strain to discriminate 30‐day mortality in patients with intermediate‐risk PE. Diastolic BP demonstrated an AUC of 0.773 (95% CI 0.482 – 1.000), while RA strain yielded an AUC of 0.751 (95% CI 0.623 – 0.879), indicating moderate discriminative performance. AUC, area under the curve; BP, blood pressure; CI, confidence interval; PE, pulmonary embolism; RA, right atrium; ROC, receiver operating characteristic.

## Data Availability

The data that support the findings of this study are available from the corresponding author upon reasonable request.
