# Supplementary material for: Right Ventricular Global Longitudinal Strain and 30‐Day Mortality in Intermediate‐Risk Pulmonary Embolism: An External Validation Study
Source: Echocardiography. 2026 Sep 10;43(9):e70612. doi: 10.1111/echo.70612 (PMC13559206; doi:10.1111/echo.70612)
Supplement: Supplementary file 1 [file ECHO-43-e70612-s001.pdf]

Table S1. Comparison of baseline clinical and echocardiographic characteristics between the derivation and external cohorts

	Derivation	External	p value
Subjects, n	251	119	-
Age, years	63.2 ± 15.8	66.1 ± 14.0	0.181
Female, n (%)	108 (43)	58 (49)	0.613
Race, Black, n (%)	20 (8)	20 (17)	0.019
Hypertension, n (%)	137 (55)	98 (82)	< 0.001
CHF, n (%)	26 (10)	34 (29)	< 0.001
CAD, n (%)	50 (20)	33 (28)	0.109
Vital sign on presentation			
Heart rate, beats/min	101.3 ± 22.2	90.6 ± 14.9	< 0.001
Systolic BP, mmHg	134.6 ± 25.8	131.8 ± 22.9	0.372
Diastolic BP, mmHg	80.9 ± 17.0	79.8 ± 16.3	0.889
Conventional echocardiographic parameters			
LVEF, %	59.8 ± 12.0	61.0 ± 10.7	0.526
LVDd, mm	45.9 ± 6.59	44.0 ± 5.93	0.010
RVDd, mm	44.0 ± 7.77	41.6 ± 6.77	< 0.001
RV/LV ratio	0.97 ± 0.17	0.95 ± 0.14	0.376
RVFAC, %	34.3 ± 8.16	33.7 ± 6.60	0.285
TAPSE, mm	18.3 ± 5.84	17.1 ± 4.19	0.035
RV S’, cm/sec	12.3 ± 4.56	12.8 ± 3.35	0.237
SPAP, mmHg	41.7 ± 15.7	43.1 ± 16.3	0.360
Strain parameters			
RVGLS, %	19.0 ± 3.71	19.5 ± 2.61	0.222
RVFWS, %	21.2 ± 4.81	21.4 ± 3.77	0.917
RA strain, %	29.9 ± 7.68	31.0 ± 6.73	0.105

Data are presented as mean ± SD or number (%), as appropriate. BP, blood pressure; CAD, coronary artery disease; CHF, chronic heart failure; LVDd, left ventricular end-diastolic diameter; LVEF, left ventricular ejection fraction; RA strain, right atrial strain; RVDd, right ventricular end-diastolic diameter; RVFAC, right ventricular fractional area change; RVFWS, right ventricular free wall strain; RVGLS, right ventricular global longitudinal strain; RV/LV ratio, right-to-left ventricular end-diastolic diameter ratio; RV S’, tissue Doppler imaging-derived right ventricular systolic velocity; SPAP, systolic pulmonary artery pressure; TAPSE, tricuspid annular plane systolic excursion. RV strain parameters are presented as absolute values.

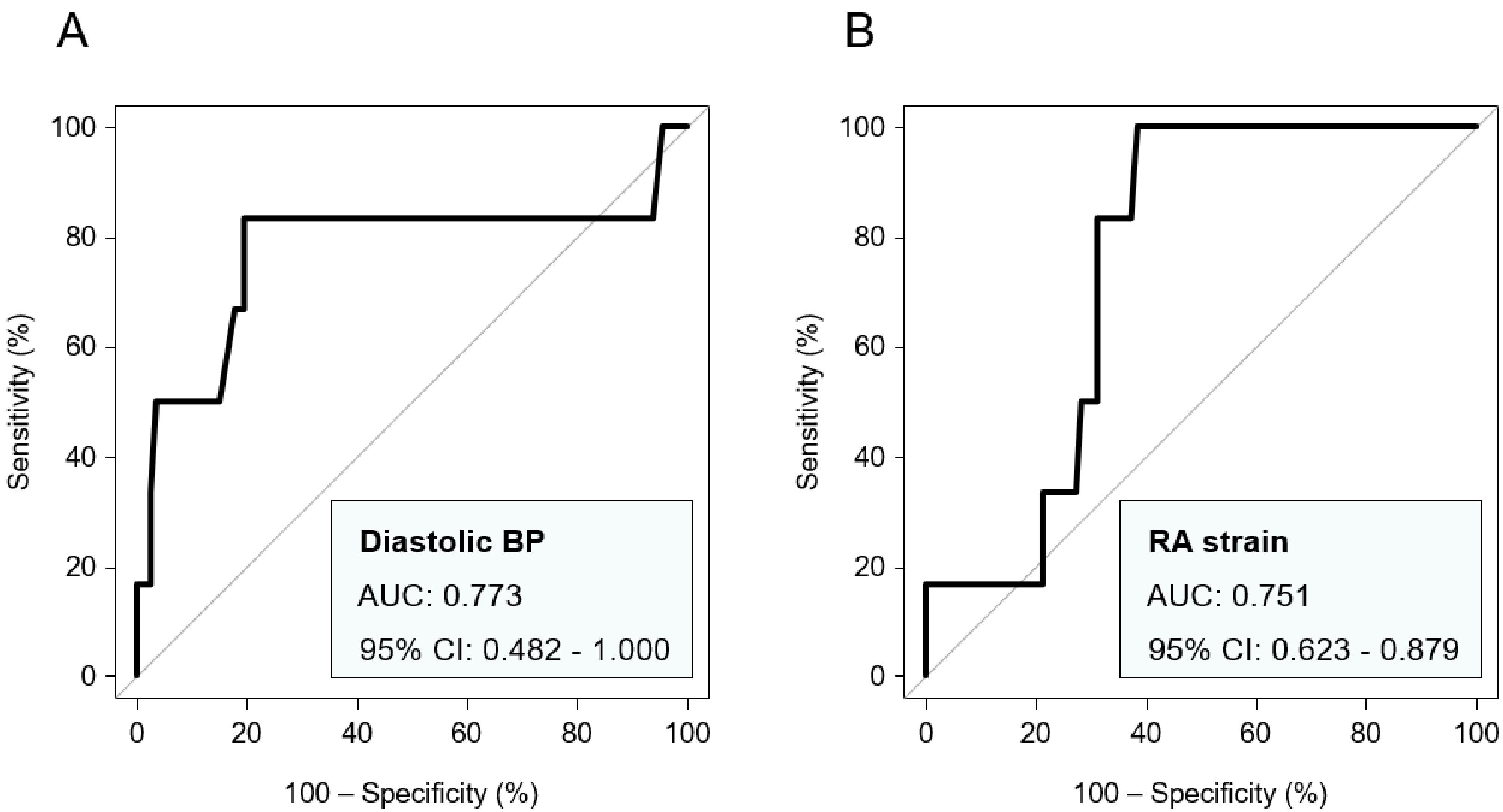

Figure S1. ROC curves of diastolic BP and RA strain for 30-day mortality. ROC analysis demonstrating the ability of (A) diastolic BP and (B) RA strain to discriminate 30-day mortality in patients with intermediate-risk PE. Diastolic BP demonstrated an AUC of 0.773 (95% CI 0.482 - 1.000), while RA strain yielded an AUC of 0.751 (95% CI 0.623 - 0.879), indicating moderate discriminative performance. AUC, area under the curve; BP, blood pressure; CI, confidence interval; PE, pulmonary embolism; RA, right atrium; ROC, receiver operating characteristic.
